# Foreign Correspondence

**Published:** 1839-08-24

**Authors:** 


### FOREIGN CORRESPONDENCE.

DUBLIN, 20*th June*, 1839.

To the Editors of the Medical Examiner.

GENTLEMEN,—The high reputation of the Dublin medical schools and of their professors, induces me to send you a few hasty observations respecting them, which I was enabled to make during a recent visit. Connected with the University, is the

*School of Anatomy*, *Trinity College*, a commodious building in the College Park, including the anatomical and chemical theatres. In the anatomical section there is a beautiful dissecting room, admirably lighted and ventilated, with a fountain and troughs in the centre. There is a private dissecting room also. The lecture room is sufficiently spacious to accommodate four hundred students; and here the anatomical, physiological, pathological, and surgical lectures are delivered. The college museum is scanty. The students have the use of a good library, on the payment of a small sum. The lectures commence on the first Monday in November, and continue till the last of May.

The *College of Physicians*, *Sir Patrick Dun's*, is similar in its objects to the same institution in London, and need not detain us.

The *Royal College of Surgeons*, on the west side of Stephen's Green, is a fine building, with a Doric façade, of mountain granite. The columns of Portland stone rest on a rustic basement, and support an elegant pediment, surmounted by three statues—Esculapius, Minerva, and Hygeia. In the tympanum of the pediment, are the royal arms. The interior is conveniently arranged, and contains a board room, for the meetings of the college; a library, with a good selection of works on medicine, surgery, and natural history; an examination hall, and numerous smaller apartments. There are also four lecture rooms, a laboratory, and several dissecting rooms. There are *three* museums. One is eighty-four feet long, by thirty broad, and thirty-six in height, with a gallery. This is appropriated to a large and well-arranged set of human and comparative anatomical preparations, wet and dry. The second museum, twenty-four feet square, and thirty-six high, with a double gallery, contains the pathological specimens, and an excellent collection of wax models, the gift of the Duke of Northumberland. The other is small, in which are the models and specimens used in the daily demonstrations. The candidates for a surgical license are obliged to study five years, to attend a certain number of courses on anatomy, physiology, surgery, chemistry, practice of medicine, materia medica, midwifery, and medical jurisprudence. They are publicly examined for two separate days, very rigorously, on all these branches. If rejected, the candidate may appeal to another court of examiners, differently constituted; and, if rejected by them, is obliged to extend his probation one year. Half yearly examinations of the registered pupils are held, and each candidate is obliged to show that he has answered four such, ere he can be eligible to the final one for his diploma. The lecture term embraces six months.

Attached to the *House of Industry*, in North Brunswick st., an immense establishment, capable of accommodating nearly two thousand *inmates*, is the *Richmond Surgical Hospital*, containing one hundred and twenty beds. The patients are admitted by the surgeons according to the urgency of the case, without reference to recommendation. The building is an ancient nunnery, and is badly adapted to its present more useful purpose, the wards being low and small. Much attention appears, however, to be paid to cleanliness and ventilation, and, considering the crowded state of the wards, with tolerable success. The operating theatre is of good size, and well lighted. The museum, commenced by Prof. Tod, of King's College, London, is indebted for its present prosperous condition to the indefatigable exertions of R. W. Smith, Esq., the accomplished surgical lecturer at the Richmond School. Although the collection cannot pretend to vie in extent with that of the College of Surgeons, it is far more valuable. It is comprised entirely of pathological specimens. The history of each case is known and registered; and there are casts and drawings of most of them, admirably executed by individuals retained exclusively for its use. Here I examined the preparations of congenital dislocations of the shoulder-joint, the subject of a novel and excellent essay, by Mr. Smith, in the last number of the Dublin Journal. Also, the specimens of rheumatic disease of the joints, so ably described in Tod's Cyclopædia, by R. Adams, Esq., of this city.

The *Pathological Society of Dublin*, promises to become a most valuable institution. Though recently established, so great has been the ardour and industry of its members, and so ample the materials, that a volume of transactions may very soon be expected. This will be illustrated by plates of the most important specimens. Each preparation has its history attached, with casts and drawings. No where in America does a more ample field exist for the establishment of a society of this description than in Philadelphia; and were it once commenced, I have not the least doubt would become one of our most cherished and important institutions. Boston already has a society of the kind, and great advantages have been found to arise from it; and, under the care of its indefatigable curator, Dr. J. B. S. Jackson, will assume before long an importance commensurate with its usefulness.

I this morning examined at Mr. Adams's, an undoubted specimen of united fracture of the neck of the femur, within the capsule. This occurred in an old female pauper, in the House of Industry, some time since. Nothing was done, and on her dying after a few months, complete ossific union was found to have taken place. The case will be detailed in the forthcoming number of the Cyclopædia of Anatomy and Physiology, *Art.* Hip-joint, by Mr. A. It is not to be regarded as contradictory of Sir Astley Cooper's views as to the rareness of reunion in such cases. In 1834, Sir Astley addressed a note to the Editor of the Medical Gazette, with the view, as he stated, of correcting an assertion of the late Baron Dupuytren's, that he (Sir Astley) had declared reunion, in cases of fracture entirely within the capsule, to be impossible. He does not deny the possibility of union, but only regards it as rare. He considers the difficulty of a false position, and not deficient nourishment, to be the obstruction to union, and adds, that where the synovial membrane is unruptured, union will occur, giving as illustrative a case of Mr. Swan's. Mr. Adams's case is exactly like that of Mr. Swan's, the rupture of the sac being limited to one side, and in that spot there is no ossific deposit. The preparation and case are highly interesting.

Mr. Adams is one of the surgeons to the Richmond Hospital, and an excellent practitioner and operator. He showed me the results of several operations for club-foot, which he had recently performed, and with success. The treatment of surgical cases is very similar to our own, though they appear to admit less readily than we do new and unestablished methods of treatment. Davat's operation for varicose veins, and Velpeau's iodine injection in hydrocele, I could not discover had yet been tried in any of their surgical hospitals. Their fracture treatment is still defective. I have had no opportunities of witnessing any operations. The same neatness and excellent ventilation, and large wards, and comfortable beds, as with us, are not seen in most of the Dublin hospitals. Great attention, however, appears to be paid the patients, and the manner of the visiting medical attendants is patient and kind.

Very respectfully yours, M. C.

